# Cognitive load alters cortical dynamics during gait in Parkinson’s disease but not in neurologically healthy individuals

**DOI:** 10.1007/s11571-026-10424-4

**Published:** 2026-02-25

**Authors:** Luis Felipe Itikawa Imaizumi, Claudiane Arakaki Fukuchi, Lucas Simieli, Carolina Rodrigues Alves Silveira, Paulo Cezar Rocha dos Santos, Sérgio Tosi Rodrigues, Paula Favaro Polastri, Fabio Augusto Barbieri

**Affiliations:** 1 Human Movement Research Laboratory (MOVI-LAB), Department of Physical Education, School of Sciences, São Paulo State University (UNESP), Bauru, SP Brazil; 2 Cognitive Neurology and Alzheimer’s Disease Research Centre, Parkwood Institute, London, ON Canada; 3 Lawson Health Research Institute, St. Joseph’s Health Care London, London, ON Canada; 4 IDOR/Pionner Science, Rio de Janeiro, RJ Brazil; 5 Laboratory of Information, Vision and Action (LIVIA), Department of Physical Education, School of Sciences, São Paulo State University (UNESP), Bauru, SP Brazil

**Keywords:** Parkinson’s disease, Dual-task, Walking, Cognition, EEG, Cortical activity

## Abstract

The level of difficulty of a secondary cognitive task (DT) can affect gait and cortical activity distinctly in individuals with Parkinson’s disease (PD). During a simpler ST, individuals with PD may use a compensatory neural mechanism by reallocating neural resources to preserve gait performance; for difficult DT, this compensation may not be the case. However, whether different levels of difficulty of a single-domain DT would distinctively affect gait and cortical activity in individuals with PD compared to neurologically healthy individuals is still unknown. Fourteen individuals with PD and 14 healthy individuals performed walking trials at self-selected speed, under six conditions of walking with an auditory DT and varying levels of difficulty (very easy: VE-SCT, easy: E-SCT, moderate: M-SCT, difficult: D-SCT, and very difficult: VD-SCT). Gait kinematics and cortical activity data were recorded. RM-ANOVAs identified that individuals with PD showed higher DT cost for both step length and step velocity when the cognitive task was D-SCT or VD-SCT, compared to easier tasks (*p* < 0.005). Cortical activity showed a different pattern. During more difficult tasks (M-SCT, D-SCT, VD-SCT), PD individuals had a lower DT cost in delta frequency (frontal and motor areas) and beta frequency (parietal area) compared to the easier tasks (VE-SCT, E-SCT) (*p* < 0.005). These findings suggest that individuals with PD exhibit a distinct pattern of cognitive-motor interaction during dual-task walking, characterized by increased cortical dual-task cost in lower vs. greater gait deterioration in higher task demands. These findings suggest that individuals with PD over-engage cognitive resources while walking with relatively easier DT.

## Introduction

Empirical evidence has shown that a secondary cognitive task negatively affects gait in individuals with Parkinson’s disease (PD) (Strouwen et al. 2016a; Vervoort et al. 2016; Yogev-Seligmann et al. 2012). Specifically, performing a secondary cognitive task while walking, i.e., dual-tasking (DT), leads to a reduction in walking speed (Maidan et al. 2016; Raffegeau et al. 2019) and decreased stride length (Maidan et al. 2016). At the neural level, individuals with PD show increased prefrontal cortex activity (Maidan et al. 2016) and beta power (global cortical analysis) (Palmer et al. 2010; Possti et al. 2021), along with decreased delta and theta power from midline centroparietal regions (Possti et al. 2021) during DT. These neural changes may differently affect motor and parietal brain areas, depending on the demands of the secondary cognitive task (Possti et al. 2021).

Cortical activity during dual-task walking in individuals with PD has been shown to differ substantially from that of neurologically healthy individuals, particularly across distinct EEG frequency bands (Orcioli-Silva et al. 2020). In general, individuals with PD tend to exhibit increased activation in frontal regions (Maidan et al. 2016), notably reflected in elevated theta and delta band activity, which are associated with attentional control and error monitoring (Cavanagh and Frank 2014; Harmony 2013). Conversely, beta-band activity, particularly in sensorimotor and occipital regions, is often suppressed during movement, and this suppression is reduced in PD (Faria et al. 2023)—indicating impaired motor planning and execution (Palmer et al. 2010). Alpha-band power, commonly associated with sensory integration and attentional inhibition, may also be altered under dual-task conditions. These spectral dynamics reflect the increasing cognitive-motor interference in PD, where shared neural resources are taxed when performing concurrent tasks.

Since individuals with PD may depend on higher-order neurological systems, such as cognitive processes (Takakusaki et al. 2023), even for basic single-task walking, their gait performance may be further impaired when walking is performed concurrently with a secondary cognitive task (Plotnik et al. 2011), potentially due to shared resources. A dysfunctional overlap between motor and cognitive processes occurs during walking with a secondary cognitive task (Pessiglione et al. 2005), leading to additional load on the frontal attentional resources that further prevents individuals with PD from effectively using compensatory frontal strategies to control gait (Orcioli-Silva et al. 2020; Wu et al. 2015). A competition for neural resources is created during DT, while the metabolism of the brain is finite (Dietrich 2006), with a limited capacity to allocate cognitive processes to perform two tasks at the same time. The ability to allocate neural resources to perform the concomitant tasks safely and effectively varies under different task demands. When this capacity is reduced, as in PD, an impaired gait performance and an increased risk for falls are evidenced (Raffegeau et al. 2019; Yogev-Seligmann et al. 2012). Nevertheless, if cognitive resources are sufficient during DT, the brain may share them between tasks without detrimental effects on either task.

Previous studies have demonstrated that the level of difficulty of a secondary task may impact gait differently in PD. In easier cognitive dual-task conditions, individuals with PD may still maintain gait performance during simpler dual-task conditions, potentially reflecting preserved capacity for integrated control under low cognitive load (Yogev et al. 2005). While executing a simpler secondary cognitive task, individuals with PD may exhibit compensatory neural engagement that supports gait control under lower cognitive demands, though this capacity may not scale with increasing task complexity. When the cognitive demands increase (i.e., more complex cognitive dual tasks) on working memory and executive functions, gait performance tends to deteriorate more substantially (Brauer and Morris 2010; Galletly and Brauer 2005; Strouwen et al. 2016b; Yogev et al. 2005). Cortical and subcortical activity seems to be highly sensitive to the difficulty of the cognitive secondary task while walking (Montero-Odasso et al. 2012a; Segev-Jacubovski et al. 2011). An unexplored question is related to whether different levels of difficulty of a cognitive secondary task (i.e., gradual change in cognitive demand) would distinctively affect gait and cortical activity in PD.

It is important to note that the use of different cognitive secondary tasks in previous research creates a challenge when trying to compare levels of difficulty across studies. For example, differences in the cognitive domain of secondary tasks (e.g., working memory vs. verbal fluency) across studies blur the conclusions due to the mixed effects of complexity and the cognitive domain of secondary tasks on gait. Proposing a single-domain cognitive secondary task (e.g., working memory) with a gradual increase in difficulty can help to fulfill this gap in the literature. In addition, most of the studies were focused on the effects of DT on gait parameters, without examining cortical activity. A broader approach that integrates cortical activity and gait parameters would offer new information regarding the underlying brain-behavior linkage during DT gait in PD.

Therefore, the purpose of the present study was to investigate whether gait and cortical activity are distinctly affected by the level of difficulty of a cognitive secondary task in PD compared to neurologically healthy individuals. We hypothesized that easier (lesser difficult working memory task) vs. more complex secondary cognitive task would reflect in lesser impact/adaptation in gait performance (lesser change in velocity and length) and cortical activation [lower changes in EEG power spectral density, mainly related to beta-band frequency domain—as this band is related to motor impairments in PD (Asadi et al. 2022; Peng et al. 2024)]. Also, we expected that the effects of walking with a more complex cognitive secondary task would be less pronounced in neurologically healthy individuals due to higher integrity of the brain system.

## Method

### Participants

Twenty-eight individuals, 14 individuals with PD and 14 neurologically healthy individuals (5 females in each group), participated in this study. The number of participants was determined by a power analysis (G*power©) based on the step velocity parameter of Bond and collaborators’ study (Bond and Morris 2000). Considering a statistical power of 95% and α of 0.05, a total sample of 9 participants per group was required.

Participants were excluded if they were younger than 60 years old, scored less than 24 on the Mini-Mental State Examination (MMSE) (Almeida 1998), had a history of neurological disorders other than PD that could affect their performance, were unable to walk independently for at least 5 min, had unstable medical conditions, including cardiovascular, hearing, and visual problems not corrected, or significant psychiatric comorbidity (i.e., self-reported history of diagnosed psychiatric conditions, such as major depressive disorder, generalized anxiety disorder, psychotic disorders).

Individuals with PD underwent a neurological evaluation, which included an anamnesis (e.g., disease duration, medication treatment), the Unified Parkinson’s Disease Rating Scale (UPDRS) (Goetz et al. 2008)d scale to assess disease severity. The MMSE and Montreal Cognitive Assessment (MoCA) were used to evaluate the general cognitive status of all participants (Romann et al. 2012). Individuals with PD were evaluated approximately 45–60 min after the first-morning dose of dopaminergic medication and considered in the ON medication state. The assessment session lasted no more than two hours to avoid the effects of the medication wearing-off.

The study was approved by the São Paulo State University (UNESP/Bauru) ethics committee (CAAE78660517.2.0000.5398) and was performed according to the principles of the Declaration of Helsinki. All participants gave their informed written consent prior to participation.

### Protocol

Participants walked while counting pre-assigned numbers spoken on an audio track. They listened to 25-second audio recordings containing about 12 numbers in random order and were told to mentally count the occurrences of one or more pre-assigned target numbers while refraining from verbal counting or using their fingers. The instructions were as follows: *“We invite you to walk at your usual*,* self-selected pace while performing the counting task as accurately as possible. In other words*,* you are going to walk while listening to an audio track containing numbers and mentally count the occurrences of one or more pre-assigned target numbers.”* No explicit task-priority instruction was given (e.g., “focus on walking” or “prioritize the cognitive task”), in order to simulate naturalistic dual-task behavior. This auditory mental-counting paradigm was selected because it imposes a controlled and progressively scalable working-memory and attentional load while minimizing speech- and manual-motor confounds, and similar counting-based dual-task paradigms have been widely used to elicit cognitive–motor interference during gait, including in PD (McPhee et al. 2022; Montero-Odasso et al. 2012b).

A new target number was given at the beginning of each trial and the participant must report the count at the end of the trial. The target number was randomized between trials. The number of times that the target number appeared in the sequence was random and ranged from 0 to 9. The gradual increase in the complexity of the cognitive task during walking was manipulated by the amount of the occurrence of target numbers (1 to 4) that participants had to count (Table 1). Thus, the difficulty of the secondary cognitive tasks increased gradually along task conditions, demanding more working memory and consequently making DT more challenging as the number of target numbers increases.

Participants performed three 25-second (uninterruptedly) trials, at self-selected velocity, for each out of six conditions of walking: (1) without cognitive secondary task (W-SCT); (2) with very easy cognitive secondary task (VE-SCT) - individuals counted the number of times a single target number appeared in an audio recording; (3) with easy cognitive secondary task (E-SCT) - individuals counted the number of times one target number appeared in an audio recording; (4) moderate cognitive secondary task (M-SCT) - individuals counted the number of times two target numbers appeared in an audio recording; (5) difficult cognitive secondary task (D-SCT) - individuals counted the number of times three target numbers appeared in an audio recording; (6) very difficult cognitive secondary task (VD-SCT) - individuals counted the number of times four target numbers appeared in an audio recording (Table 1). Individuals performed a total of 18 trials, and the order of conditions was randomized. A resting period of 1 min was given between conditions to avoid fatigue.

Prior to performing the walking conditions and to calculating the DT cost, participants performed two trials while sitting for each DT condition (randomized order). They only listened to the audio recording and the counting accuracy was measured. Accuracy during the cognitive task (seated and walking) was calculated by absolute error: correct answer—participant answer (e.g., correct answer: 12; participant answer: 10—absolute error = 2). Finally, individuals performed one 5-second trial seated without any task, which was used to normalize cortical activity. Participants were instructed to avoid any thoughts during this period.

**Table 1:** Study conditions according to difficulty. An example of the correct answer for each condition was shown in the last column

Condition	Audio recording	Target number	Answer
Very easy secondary task (VE-SCT)	1 1 1 1 1 1 1 1 1 1 1 1	1	12
Easy secondary task (E-SCT)	0 2 8 4 1 1 5 6 2 1 2 9	1	3
Moderate secondary task (M-SCT)	8 2 2 4 1 1 9 7 2 1 2 5	1; 2	7
Difficult secondary task (D-SCT)	7 2 2 4 8 1 5 9 2 1 2 5	1; 2; 5	8
Very difficult secondary task (VD-SCT)	0 2 9 4 1 7 5 6 2 1 2 8	1; 2; 5; 6	7

### Kinematics and cortical activity recording and processing

Gait kinematics were obtained from 10 cameras (Vicon Motion System^®^, Oxford, England) at 200 Hz. Thirty-nine passive reflective markers were placed according to the Plug-in-Gait Full Body model (Vicon Motion System^®^, Oxford, England). However, only the markers at the second metatarsal and the heel were used to calculate gait parameters. Based on previous studies, the data were filtered using a 5th-order low-pass digital Butterworth filter (zero-lag) with a cutoff frequency of 6 Hz (Penedo et al. 2018). Gait parameters were calculated using Matlab (version R2016b).

Step length, width, duration, and velocity, and double support time (as a percentage of step duration) were calculated. Spatial-temporal parameters were calculated using 10 to 15 steps from each trial, removing the acceleration and deceleration phase of walking (the first and last two steps of each trail).

Cortical activity data were recorded from 64 active electrodes electroencephalogram (eegosportstm, ANT Neuro, Enschede, Netherlands; 1024 samples/s), a wireless, wearable amplifier. The cap (WaveGuard™) and the electrode position followed the 10–10 International system electrode placement (Oostenveld and Praamstra 2001) and manufacturers’ recommendations (ANT Neuro, Enschede, Netherlands). Participants carried the amplifier in a lightweight backpack specifically designed for mobile EEG acquisition during movement. The impedance remained below 10 Ω on the electrodes. Signal data obtained from all 64 electrodes were imported into the EEGlab environment (Delorme et al. 2011). The data was filtered with a band-pass filter (cut-off frequency 0.5–50 Hz) and visually inspected to identify large artifact periods (Whittier et al. 2020). Channels with a standard deviation higher than 400 µV and above five standard deviations from the mean were removed (https://sccn.ucsd.edu/wiki/EEGLAB_Wiki) (Gwin et al. 2010). A down-sample was performed to 512 Hz and the data were referenced to the signal average (Santinelli et al. 2021). Independent Component Analysis (ICA- Runica) was used to remove additional artifacts, such as eye movements, blinks, muscle activity of the facial muscles, and other potential artifacts (such as heart activity) (Radüntz et al. 2015).

The average power spectral density (PSD) was determined considering delta: 0.5–3 Hz, theta: 4–7 Hz, alpha: 8–12 Hz, and beta: 13–30 Hz frequency bands (Santinelli et al. 2021). These various cortical activity measures have been strongly related to mental effort, attention allocation, readiness, and arousal (Berka et al. 2007). Three regions of interest were determined for the cortical analysis: frontal (F3, F4 and Fz), motor (C3, C4 and Cz), and parietal (P3, P4 and PZ) (Orcioli-Silva et al. 2025). To exclude the possibility of divergence in the cortical activity analysis, only one researcher performed all procedures. Cortical activity measurements during experimental conditions were normalized based on cortical activity while sitting without any assigned task (Oliveira et al. 2016).

Finally, we calculated the DT cost (Maidan et al. 2019) for gait and cortical activity parameters:

$$[({\mathrm{DT}}\,{\mathrm{condition}} - {\mathrm{walking}}\,{\mathrm{without}}\,{\mathrm{DT}})/{\mathrm{walking}}\,{\mathrm{without}}\,{\mathrm{DT}}] \times {\mathrm{1}}00$$

### Statistical analysis

Statistical analysis was performed using SPSS version 25. Statistical significance was set to *p* = 0.05. Data were normally distributed, and parametric statistics were therefore used. Cognitive scales (MMSE and MoCA) and demographic characteristics (age, height, body mass) were compared between groups using t-test. Mixed two-way ANOVAs with between-group (2 levels: PD and control) and within-conditions (6 levels: W-SCT, VE-SCT, E-SCT, M-SCT, D-SCT and VD-SCT) levels were used to identify differences in gait and cortical activity parameters. Mixed two-way ANOVAs with between-group and within-conditions (5 levels: VE-SCT, E-SCT, M-SCT, D-SCT, and VD-SCT) were used to examine differences in absolute error and DT cost for gait and cortical activity parameters. Partial eta-squared $\:\left({\eta\:}_{p}^{2}\right)$ was reported to measure effect size and interpreted as small (> 0.01), moderate (> 0.06) or large (> 0.14) effect (Cohen 2013). Tukey post hoc tests, with p-adjusted according to the number of comparisons (i.e., 6 levels: *p* < 0.003; 5 levels: *p* < 0.005), were used when a significant main or interaction effect was found. Intraclass correlation coefficient (ICC) was taken as a reliability estimate of cortical activity measurements across trials and was interpreted as being poor (< 0.50), moderate (0.50–0.75), good (0.75–0.90), or excellent (> 0.90).

## Results

Participants’ characteristics are presented in Table 2. There was no significant difference between groups for age, height, body mass, MMSE, and MoCA.

**Table 2:** Participants’ characteristics. Values are expressed as mean ± standard deviation and the range is presented in brackets

Age (years)	Individuals with PD	Neurologically healthy individuals	*p*-values
	69 ± 5 [61–80]	67 ± 5 [59–79]	0.36
Height (m)	1.66 ± 0.07 [1.56–1.76]	1.65 ± 0.10 [1.49–1.84]	0.76
Body mass (kg)	74.8 ± 15.2 [49–97]	75.6 ± 12.5 [59–94]	0.87
MMSE (pts)	28.2 ± 1.1 [27–30]	27.8 ± 2.2 [24–30]	0.59
MoCA (pts)	27.2 ± 1.5 [24–30]	26.7 ± 1.6 [24–28]	0.35
UPDRS motor (pts)	29.2 ± 0.3 [18–58]	–	–
H&Y scale (pts)	2.2 ± 0.3 [2–3]	–	–

MMSE Mini-mental state exam; MoCA Montreal Cognitive Assessment; UPDRS Unified Parkinson’s Disease Rating Scale; H&Y Hoehn & Yahr

### Absolute error during seated and walking tasks

**Fig. 1:**
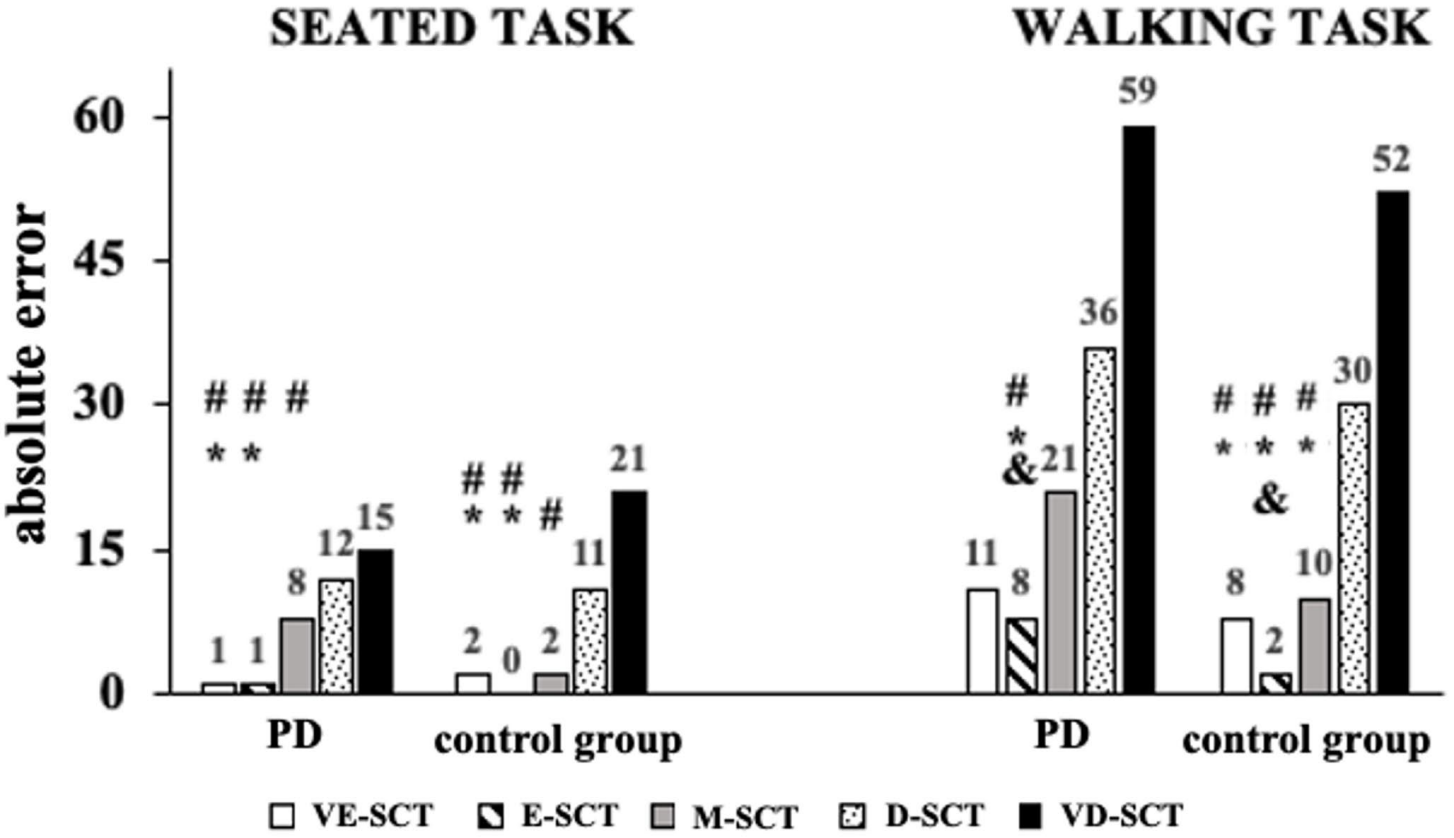
Sum of absolute error in the cognitive secondary task for each condition during seated and walking tasks in individuals with PD (PD patients) and neurologically healthy individuals (control group). The numbers above each bar represents the absolute error. * - significant different from D-SCT; # - significant different from VD-SCT; & - significant different from M-SCT. *Note: very easy secondary task (VE-SCT)*,* easy secondary task (E-SCT)*,* moderate secondary task (M-SCT)*,* difficult secondary task (D-SCT)*,* very difficult secondary task (VD-SCT)*

Absolute error during seated and walking conditions is presented in Fig. 1. For both seated and walking tasks, there were no significant main effects of group (F_1,26_ = 0.14; *p* = 0.91 and F_1,26_ = 1.30; *p* = 0.26, respectively) nor group*condition interaction (F_4,104_ = 1.65; *p* = 0.16 and F_4,104_ = 0.102; *p* = 0.98, respectively).

A main effect of condition was observed for both seated (F_4,104_ = 19.26; *p* < 0.001; $\:{\eta\:}_{p}^{2}$= 0.42 *[large]*) and walking tasks (F_4,104_ = 21.11; *p* < 0.001; $\:{\eta\:}_{p}^{2}$= 0.44 *[large]*). During the seated task, D-SCT showed greater absolute error than VE-SCT and E-SCT (*p* < 0.001). Also, participants had greater absolute error during VD-SCT compared to VE-SCT, E-SCT, and M-SCT (*p* < 0.001). During the walking task, individuals had greater absolute error in M-SCT, D-SCT, and VD-SCT compared to E-SCT (*p* < 0.001). In addition, absolute error was greater in D-SCT and VD-SCT compared to VE-SCT (*p* < 0.001) and M-SCT (*p* < 0.003).

### Gait parameters

#### Average values

**Fig. 2:**
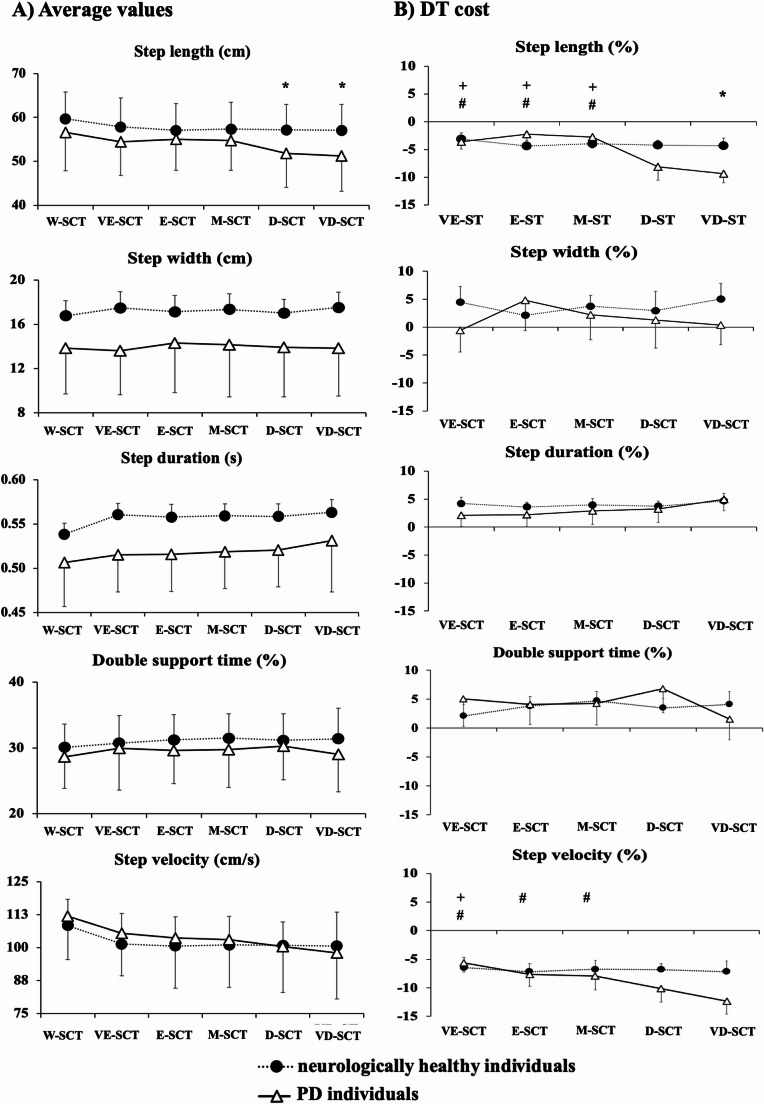
(A) Means d standard deviations of kinematic gait parameters for individuals with PD and neurologically healthy individuals. (B) means and standard errors of DT cost of kinematic gait parameters for individuals with PD and neurologically healthy individuals. * - individuals with PD significant different from neurologically healthy individuals, + - siangnificantly different from D-SCT in PD individuals; **#** - significantly different from VD-SCT in PD individuals. *Note: without secondary task (W-SCT)*,* very easy secondary task (VE-SCT)*,* easy secondary task (E-SCT)*,* moderate secondary task (M-SCT)*,* difficult secondary task (D-SCT)*,* very difficult secondary task (VD-SCT)*

Mixed ANOVAs revealed a main effect of group for step duration (F_1,26_ = 4.95; *p* < 0.03; $\:{\eta\:}_{p}^{2}\:$= 0.16) (Fig. 2A), and a main effect of condition for step length (F_5,130_ = 10.18; *p* < 0.001; $\:{\eta\:}_{p}^{2}$ = 0.28 *[large]*), step duration (F_5,130_ = 4.74; *p* < 0.005; $\:{\eta\:}_{p}^{2\:}$= 0.15 *[large]*) and step velocity (F_5,130_ = 20.19; *p* < 0.001; $\:{\eta\:}_{p}^{2}\:$= 0.43), and a group*condition interaction for step length (F_5,26_ = 2.89; *p* < 0.04; $\:{\eta\:}_{p}^{2}\:$= 0.10).

In general, individuals with PD had shorter step duration compared to neurologically healthy individuals. Independent of condition, participants walked with a larger step length in W-SCT compared to D-SCT and VD-SCT conditions (*p* < 0.001), shorter step duration in W-SCT compared to VD-SCT condition (*p* < 0.001), and faster step velocity in W-SCT compared to all conditions with DT (*p* < 0.001).

Post hoc analysis indicated that individuals with PD walked with shorter step length when performing walking with D-SCT and VD-SCT compared to neurologically healthy individuals (*p* < 0.003).

#### DT cost

Mixed ANOVAs revealed a main effect of condition for DT cost of step length (F_4,104_ = 3.79; *p* < 0.03; $\:{\eta\:}_{p}^{2}\:$= 0.12) and velocity (F_4,104_ = 4.08; *p* < 0.01; $\:{\eta\:}_{p}^{2}\:$= 0.13), and a significant group*condition interaction for DT cost of step length (F_4,26_ = 3.06; *p* < 0.05; $\:{\eta\:}_{p}^{2}\:$= 0.10) and velocity (F_4,26_ = 3.01; *p* < 0.03; $\:{\eta\:}_{p}^{2}\:$= 0.11). No significant main effect of group for DT cost.

Independent of condition, DT cost of step length (*p* < 0.005) and velocity (*p* < 0.002) increased when the individuals performed walking with VD-SCT compared to VE-SCT (Fig. 2B).

Post hoc analysis indicated that individuals with PD showed higher DT cost of step length when walking with VD-SCT compared to neurologically healthy individuals (*p* < 0.004). Within the PD group, DT cost of step length was significantly higher during walking with D-SCT and VD-SCT compared to VE-SCT, E-SCT, and M-SCT (*p* < 0.005 and *p* < 0.002, respectively). Similarly, performing walking with VD-SCT increased the DT cost of step velocity compared to VE-SCT, E-SCT, and M-SCT (*p* < 0.001). Additionally, the DT cost of step velocity was higher in D-SCT compared to VE-SCT (*p* < 0.005). No significant effects were observed in neurologically healthy individuals.

### Cortical activity parameters

#### Average values

ICC across trials values ranged from 0.89 *[moderate]* to 0.99 *[excellent]*. No significant main effect of group and condition, nor interaction effects for average values of cortical activity for frontal, motor, and parietal areas were found (Table 3).

#### DT cost

Mixed ANOVAs indicated (i) a main effect of group for frontal (delta and alpha frequencies – F_1,26_ = 14.45; *p* < 0.001; $\:{\eta\:}_{p}^{2}\:$= 0.35 *[large]* and F_1,26_ = 6.69; *p* < 0.01; $\:{\eta\:}_{p}^{2}\:$= 0.20 *[large]*, respectively), motor (delta and alpha frequencies – F_1,26_ = 8.91; *p* < 0.006; $\:{\eta\:}_{p}^{2}\:$= 0.25 *[large]* and F_1,26_ = 8.10; *p* < 0.008; $\:{\eta\:}_{p}^{2}\:$= 0.23 *[large]*, respectively) and parietal (delta, alpha, and beta frequencies – F_1,26_ = 4.18; *p* < 0.05; $\:{\eta\:}_{p}^{2}\:$= 0.13 *[moderate]*; F_1,26_ = 4.35; *p* < 0.04; $\:{\eta\:}_{p}^{2}\:$= 0.14 *[moderate]*; and – F_1,26_ = 8.91; *p* < 0.006; η² = 0.25 *[large]* and F_1,26_ = 7.76; *p* < 0.01; $\:{\eta\:}_{p}^{2}\:$= 0.23 *[large]*, respectively) areas, and (ii) a main effect of condition for DT cost in frontal (delta and beta frequencies – F_4,104_ = 3.63; *p* < 0.01; $\:{\eta\:}_{p}^{2}\:$= 0.12 *[moderate]* and F_4,104_ = 2.91; *p* < 0.03; $\:{\eta\:}_{p}^{2}\:$= 0.10 *[moderate]*, respectively), motor (delta, theta and beta frequencies – F_4,104_ = 5.30; *p* < 0.001; $\:{\eta\:}_{p}^{2}\:$= 0.17 *[large]*, F_4,104_ = 4.30; *p* < 0.004; $\:{\eta\:}_{p}^{2}\:$= 0.14 *[moderate]* and F_4,104_ = 3.09; *p* < 0.03; $\:{\eta\:}_{p}^{2}\:$= 0.11 *[moderate]*, respectively), and parietal (alpha and beta frequencies – F_4,104_ = 4.07; *p* < 0.008; $\:{\eta\:}_{p}^{2}\:$= 0.13 *[moderate]* and F_4,104_ = 4.91; *p* < 0.007; $\:{\eta\:}_{p}^{2}\:$= 0.15 *[large]*, respectively) areas, and (iii) a group*condition interaction for DT cost in frontal (delta and theta frequencies – F_4,26_ = 2.68; *p* < 0.05; $\:{\eta\:}_{p}^{2}\:$= 0.09 *[moderate]* and F_4,26_ = 4.23; *p* < 0.008; $\:{\eta\:}_{p}^{2}\:$= 0.14 *[moderate]*, respectively), motor (delta and theta frequencies – F_4,26_ = 3.24; *p* < 0.02; $\:{\eta\:}_{p}^{2}\:$= 0.11 *[moderate]* and F_4,26_ = 4.23; *p* < 0.007; $\:{\eta\:}_{p}^{2}\:$= 0.14 *[moderate]*, respectively) and parietal (alpha and beta frequencies – F_4,26_ = 4.31; *p* < 0.006; $\:{\eta\:}_{p}^{2}\:$= 0.14 *[moderate]* and F_4,26_ = 2.83; *p* < 0.05; $\:{\eta\:}_{p}^{2}\:$= 0.09 *[moderate]*, respectively) areas.

In general, individuals with PD showed *largely* higher DT costs of cortical activity compared to neurologically healthy individuals for all cortical areas and frequencies (all *p* < 0.05). Independent of condition, pairwise comparisons only showed significant differences for delta frequency of the motor area when individuals performed walking with E-SCT compared to M-SCT, D-SCT, and VD-SCT (*p* < 0.005).

Post hoc analysis indicated that PD individuals had higher DT cost than neurologically healthy individuals during walking for: (i) delta frequency of frontal and motor areas and beta frequency of parietal area for VE-SCT and E-SCT (*p* < 0.005), (ii) alpha of parietal area for E-SCT (*p* < 0.004), and (iii) theta frequency of frontal and motor areas for M-SCT conditions (*p* < 0.002). For PD, participants walked during M-SCT, D-SCT, and VD-SCT with lower DT cost for: (i) delta frequency of frontal and motor areas and beta frequency of parietal area compared to VE-SCT (*p* < 0.005), (ii) delta frequency of frontal and motor areas and alpha and beta frequencies of parietal area compared to E-SCT (*p* < 0.002). Also, individuals with PD walked with lower DT cost for the alpha frequency of the parietal area for M-SCT compared to VE-SCT (*p* < 0.004). We found no significant effects for neurologically healthy individuals.

**Table 3:** Means and standard deviations of frontal, motor and parietal cortices activity according to frequencies (delta, theta, alpha and beta) and DT difficult in individuals with PD and neurologically healthy individuals

Area	Frequency	Individuals with PD						Neurologically healthy individuals					
		W-SCT	VE-SCT	E-SCT	M-SCT	D-SCT	VD-SCT	W-SCT	VE-SCT	E-SCT	M-SCT	D-SCT	VD-SCT
Frontal (µv^2^/Hz)	Delta	3.3 ± 4.6	2.1 ± 2.4	3.3 ± 3.4	3.2 ± 3.9	3.8 ± 3.4	2.9 ± 2.2	3.7 ± 3.3	3.4 ± 3.0	4.1 ± 4.1	3.7 ± 3.6	3.8 ± 4.0	3.7 ± 3.7
	Theta	2.1 ± 2.0	2.1 ± 2.0	2.0 ± 2.1	1.8 ± 1.6	2.0 ± 1.9	1.9 ± 1.8	2.1 ± 1.4	1.8 ± 1.1	1.9 ± 1.1	1.9 ± 1.1	1.8 ± 1.1	1.9 ± 1.0
	Alpha	1.1 ± 1.0	1.1 ± 0.9	1.3 ± 1.3	1.1 ± 1.0	1.4 ± 1.2	1.2 ± 1.3	1.4 ± 1.4	1.4 ± 1.1	1.4 ± 1.3	1.3 ± 1.2	1.3 ± 1.2	1.3 ± 1.2
	Beta	1.0 ± 0.i	1.0 ± 0.9	1.3 ± 1.4	1.1 ± 0.9	1.0 ± 0.7	0.9 ± 0.5	1.6 ± 1.3	1.6 ± 1.0	1.6 ± 1.2	1.7 ± 1.1	1.7 ± 1.2	1.5 ± 1.0
Frontal (µv^2^/Hz)	Delta	8.2 ± 12.9	5.2 ± 5.8	5.7 ± 6.2	7.3 ± 10.8	6.3 ± 7.7	9.5 ± 10.2	4.5 ± 4.7	4.8 ± 5.2	5.5 ± 6.1	4.9 ± 5.3	5.2 ± 5.5	4.7 ± 4.4
	Theta	3.0 ± 4.3	2.7 ± 4.4	2.9 ± 5.0	2.6 ± 4.1	3.2 ± 4.7	2.9 ± 5.1	2.9 ± 3.1	2.5 ± 2.8	2.3 ± 2.2	2.5 ± 2.4	2.5 ± 2.4	2.3 ± 2.0
	Alpha	2.0 ± 3.4	1.3 ± 1.4	2.7 ± 5.1	1.4 ± 1.8	2.3 ± 4.1	0.9 ± 1.0	1.8 ± 1.4	1.9 ± 1.7	1.6 ± 1.3	1.7 ± 1.2	1.7 ± 1.4	1.6 ± 1.3
	Beta	1.5 ± 1.9	1.5 ± 1.8	1.1 ± 0.9	1.5 ± 2.0	1.6 ± 2.1	1.2 ± 0.8	2.3 ± 2.0	2.0 ± 1.7	2.0 ± 1.7	2.0 ± 1.6	2.1 ± 1.8	1.8 ± 1.5
Frontal (µv^2^/Hz)	Delta	7.5 ± 16.6	4.0 ± 6.6	4.4 ± 7.0	3.3 ± 4.7	4.4 ± 7.1	12.1 ± 24.3	6.4 ± 10.3	5.6 ± 9.3	6.3 ± 10.4	6.0 ± 10.1	5.0 ± 9.6	5.7 ± 9.2
	Theta	2.1 ± 2.4	1.7 ± 1.9	2.2 ± 3.0	1.7 ± 2.0	2.2 ± 2.8	2.0 ± 2.6	2.4 ± 2.1	2.3 ± 2.7	1.8 ± 1.2	2.0 ± 1.4	2.5 ± 2.8	2.0 ± 1.4
	Alpha	1.6 ± 2.2	1.2 ± 1.5	1.5 ± 1.9	1.5 ± 2.0	1.6 ± 2.3	1.4 ± 1.7	1.8 ± 1.9	2.6 ± 4.9	1.7 ± 1.8	1.6 ± 1.5	1.8 ± 2.0	1.6 ± 1.5
	Beta	1.0 ± 0.8	1.1 ± 1.0	1.4 ± 1.6	1.0 ± 0.8	1.0 ± 0.8	1.0 ± 1.0	2.2 ± 1.8	1.9 ± 1.4	1.9 ± 1.4	1.8 ± 1.4	2.1 ± 1.9	1.7 ± 1.3

**Fig. 3:**
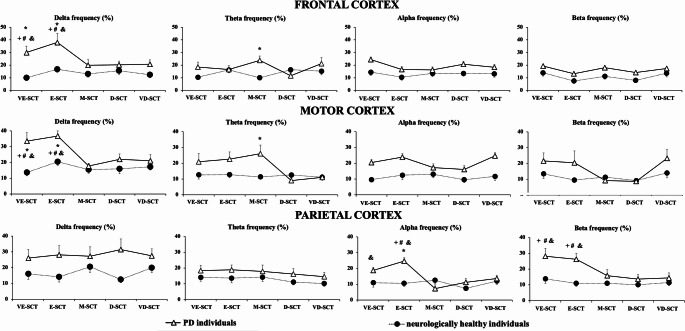
Means and standard deviations of DT cost for frontal (first line), motor (second line) and parietal (third line) cortices activity according to frequencies (delta, theta, alpha and beta) and DT difficult in individuals with PD and neurologically healthy individuals. * - individuals with PD significant different from neurologically healthy individuals; & - significantly different from M-SCT in PD individuals; + - significantly different from D-SCT in PD individuals; # - significantly different from VD-in PD individuals. Very easy secondary task (VE-SCT), easy secondary task (E-SCT), moderate secondary task (M-SCT), difficult secondary task (D-SCT), very difficult secondary task (VD-SCT)

## Discussion

To our knowledge, this is the first study that specifically manipulated a gradual increase in levels of difficulty of a single-domain cognitive secondary task (e.g., working memory) to investigate whether gait and cortical activity are distinctly affected in individuals with PD compared to neurologically healthy individuals. Our results partially supported our hypothesis. While increasing the level of cognitive difficulty tasks led to the expected increase in absolute errors, indicating that our protocol worked, changes in cortical activity and gait were selectively distinct from the original hypotheses and group-specific. The group-specific DT cost effects in cortical activation were mainly observed in easier vs. more difficult tasks, with minimal specific group-by-condition interaction in gait outcomes. Our results suggest task prioritization for individuals with PD by indicating that when the complexity of the secondary cognitive task was too high, they were unable to allocate cortical resources into both tasks, prioritizing gait over cognitive performance. We discuss first the potential PD-related factors impairing cortical control of gait and then PD-specific modulation to dual task conditions.

### PD-related impairments in neural control of dual-task walking

Our results revealed that impairments in cortical control of gait in individuals with PD emerged primarily during dual-task walking, as reflected in the DT cost, rather than in absolute measures of cortical activation. Unexpectedly, when comparing mean cortical activation across walking conditions (W-SCT, VE-SCT, E-SCT, M-SCT, D-SCT, and VD-SCT), we found no significant main effect of group in any frequency band (Table 3). This contrast with previous findings that report altered cortical activity in PD even during habitual single-task walking (Takakusaki et al. 2023) as well as under dual-task conditions (Plotnik et al. 2011). One possible explanation for the absence of a group-level effect may lie in the averaging approach used across multiple task conditions. Combining cortical data from varying task complexities could have attenuated or masked specific condition-related effects, especially if those effects differ in direction or magnitude across tasks. Additional explanation may include “pooling” effects, heterogeneity of the population, and sample size. While pooling across task conditions may have contributed to the attenuation of between-group effects, we acknowledge that other factors, including inter-individual variability, limited sample size, and potential measurement limitations, may have also contributed to the lack of significant findings in some comparisons. Additionally, EEG responses in ambulatory settings are inherently variable, and subtle group differences may have been obscured without a larger sample or more sensitive analysis methods (e.g., source localization or trial-level modeling).

The above-described argument may also explain the group’s main effect, indicating the unexpectedly shorter step duration in PD (Fig. 2A). Such results are in opposition to cumulative evidence indicating longer instead of shorter step duration in PD vs. neurologically healthy individuals, which is almost always combined with other additional changes in gait metrics, e.g., shorter and slower steps/strides (Maidan et al. 2016). Step duration represents the temporal aspect of gait control, reflecting the time elapsed between initial foot contact and the subsequent step. In PD, longer durations have been linked to cautious gait and instability, enhancing risk of falls (Berardelli 2001). It is classically attributed to PD typical brady and hypokinesia. However, while frequently longer step durations are combined with short lengths, the clinical meaning of reduced duration alone, as it was the case here, is counterintuitive. Presumably, the shorter step duration observed in our PD group may suggest a maladaptive response under increased attentional load, potentially due to difficulty coordinating motor planning and execution during dual-task walking. It is thus reasonable that the combination of multiple tasks diluting the effects of EEG is likely to minimize the potential group differences in gait performance.

### Complexity of secondary task and PD-specific adjustment in cortical control of gait

Our results partially supported the initial hypothesis. As expected, increasing the difficulty of the secondary task was associated with a higher number of cognitive errors in both PD and neurologically healthy individuals, during both seated and walking conditions. We agree that the increase in cognitive errors and gait deterioration with increased task complexity reflects expected dual-task interference. However, these results were essential to validate our novel, graded cognitive task and interpret the divergence between neural and behavioral outcomes. While the behavioral findings confirm task sensitivity, the more novel insight lies in the mismatch between EEG and gait effects across complexity levels, highlighting dynamic compensatory shifts in PD. Changes in cortical activation and gait performance emerged in a group-specific manner—primarily in individuals with PD—and were most pronounced during relatively easier DT conditions. We interpret these findings as evidence that, due to limitations in cognitive capacity, individuals with PD are only able to engage fully in the secondary task during walking when the cognitive load is relatively low. In these easier DT conditions (e.g., VE-SCT and E-SCT), PD individuals exhibited greater DT cost in parietal cortical activity, particularly in the alpha and beta bands, which are associated with sensory-motor integration and motor control (Fig. 3).

Interestingly, when task complexity increased, individuals with PD made more cognitive errors (Fig. 1), yet exhibited reduced cortical activity. This pattern may reflect limitations in the capacity to sustain integrated cognitive–motor control under increasing task demands in PD, rather than a strategic reallocation of resources. Specifically, the reduced cortical engagement observed during more complex tasks likely reflects a failure to scale neural integration when cognitive load exceeds system capacity. This interpretation is consistent with conceptual frameworks describing constrained control of gait under dual-task conditions in PD (Bloem et al. 2006). In contrast, neurologically healthy individuals did not exhibit such distinct modulation in cortical responses across task complexities. This interpretation is further supported by specific changes in cortical activation frequencies and regions. Delta-band is often associated with effortful attention and error monitoring (Cavanagh and Frank 2014; Harmony 2013). Increased delta-band activity in frontal and motor regions (Fig. 3) may confirm that relatively easier secondary cognitive tasks reflected in attentional engagement of the frontal region, which may result in inefficient gait control via motor region. Taken together, these results suggest that in PD, easier cognitive tasks during walking impose a greater neural burden due to the need to consciously regulate both gait and cognitive processing simultaneously (Bloem et al. 2006). In addition to delta-band effects, we observed significant condition-specific modulation in alpha and beta bands, particularly in the parietal region. Increased DT cost in alpha power in PD during easier tasks (VE-SCT and E-SCT) may reflect greater attentional engagement and sensory gating demands, as alpha suppression has been linked to selective attention and task-relevant inhibition (Foxe and Snyder 2011). Similarly, changes in beta-band activity, especially in the parietal area, are interpreted as markers of motor planning and cognitive-motor interference (Tzagarakis et al. 2010). The elevated beta DT cost in PD during lower-complexity tasks likely reflects the high neural effort required for motor control under dual-task conditions.

One of the most striking patterns in our data is the divergent emergence of group differences in EEG vs. gait outcomes: group effects in EEG were most pronounced during low complexity tasks (VE-SCT, E-SCT), whereas gait differences were strongest during great complexity tasks (D-SCT, VD-SCT). This pattern aligns with prior findings indicating that spectral power across multiple bands (delta, theta, alpha, beta) is sensitive to dual-task walking in PD and aging populations, possibly reflecting dynamic adjustments in neural resource allocation when cognitive load increases (Possti et al. 2021). The stronger EEG modulation during easier dual tasks may represent a front-loaded compensatory response, where cognitive and sensorimotor networks are engaged to maintain gait performance. As task complexity increases and cognitive capacity is exceeded, neural engagement appears to drop, and gait performance deteriorates—consistent with known dual-task gait decrements in PD under high cognitive load (Possti et al. 2021). Regionally, the pronounced effects in parietal alpha and beta bands, alongside frontal/motor delta and theta bands, are compatible with the functional interpretation of these rhythms: parietal alpha/beta modulating attention and sensorimotor integration, and frontal/motor lower-frequency bands mediating motor planning and attentional control under dual-task demands (Huang et al. 2024; Foxe and Snyder 2011).

When interpreted alongside gait parameters, the interpretation of DT affecting mainly PD is further supported. We observed that PD vs. neurologically healthy individuals walked with shorter step lengths, specifically during more complex dual-task conditions (D-SCT and VD-SCT—Fig. 2). Additionally, our results indicated that DT cost in both step length and step velocity was significantly greater in PD—particularly during the VD-SCT condition—indicating a more substantial motor performance decline under high cognitive demand (Fig. 2). Critically, these gait changes were not observed in neurologically healthy individuals, underscoring a PD-specific vulnerability to cognitive-motor interference (Raffegeau et al. 2019). This idea agrees with empirical evidence showing that individuals with PD vs. controls are primarily affected when they need to walk while performing a secondary cognitive task (Vervoort et al. 2016; Yogev-Seligmann et al. 2012). On the other hand, neurologically healthy individuals exhibited no DT cost differences across varying task difficulties. This finding indicates that the capacity to allocate neural resources to execute concurrent tasks remains unaffected in this cohort. Consequently, they are capable of allocating sufficient attention to a secondary task without substantially compromising their gait or balance, even during more challenging cognitive DT tasks.

These patterns indicate that in PD, the cognitive and motor systems are not simultaneously scalable across increasing dual-task demands. In simpler tasks, cognitive systems are recruited at a high cost, while in more complex tasks, motor systems show decline, presumably because cognitive resources are limited in PD. This complementary evidence suggests a dynamic resource trade-off in PD, with task-dependent shifts between neural activation and motor performance. Our results suggest that the effects of task difficulty level were not linear, resulting in a threshold effect at moderate difficulty in the secondary task. While an easy task was not challenging enough to change the DT cost, a more difficult task was so demanding that mainly PD individuals reached their limit in terms of cognitive and motor performance. Thus, at more difficult tasks, PD individuals seem to emphasize motor function, potentially as a protective strategy to mitigate fall risk, one of the major impairments in this population (Allen et al. 2013; Creaby and Cole 2018). Therefore, in practical settings, health professionals and trainers should select an adequate level of secondary cognitive task to train gait with DT in individuals with PD, modulating the brain activity to a more engaged and efficient rehabilitation.

### Strengths and limitations

A strength aspect of our study was that we were able to gradually manipulate the difficulty level of the cognitive secondary task. Our results of absolute error confirmed that the level of difficulty of the cognitive secondary task increased gradually in both seated and walking tasks. However, we should call attention to the lack of a significant difference in absolute error between D-SCT and VD-SCT, which may suggest that D-SCT has already reached the limit of cognitive capacity in PD and neurologically healthy individuals. A second strength of our study was the use of a single-domain cognitive task, avoiding conflicting results when different cognitive domains are compared, and increasing the reliability of our findings. Finally, the comprehensive combination of gait parameters and cortical activity analysis extends previous works by exploring alterations in both motor and brain systems, improving our understanding of the DT effects on walking in individuals with PD.

Some limitations in this study should be acknowledged. First, the sample size of PD individuals limited our analysis to further investigate the PD based on the progression of the disease. Since it is known that, depending on the disease severity, cognition can be more or less affected, having groups with different levels of PD could enhance our understanding of the cognitive impairment. Second, although the PD individuals were tested during the ‘ON’ state as a ‘safety’ window for them to perform the task, the medication effect could also have diminished the effects of DT difficulty. Thus, future studies with a greater sample size, a variety of PD levels of severity, and assessments during the ‘OFF’ state should be conducted to enhance our understanding of the PD population. In addition, the EEG analysis was conducted at the channel level, where activity from three electrodes was grouped to represent each cortical region (e.g., Cz, C3, C4 for motor cortex). While this approach provides a regional overview, it lacks the spatial precision of source-level analyses, which could help dissociate overlapping neural sources and improve anatomical specificity. Future studies using source localization techniques (e.g., sLORETA or beamforming) may yield more detailed insights into cortical generators involved in dual-task walking. Finally, although significant results were observed in several neural and gait parameters, the study included multiple comparisons across conditions, frequency bands, and regions, which may have inflated the risk of Type I error. Despite using standard thresholds for statistical significance, the limited sample size further constrained statistical power, raising the possibility of underpowered or spurious effects.

## Conclusion

This study reveals that individuals with PD exhibit a distinct pattern of cognitive-motor interaction during dual-task walking, characterized by increased cortical dual-task cost in lower vs. greater gait deterioration in higher task demands. These findings suggest that individuals with PD over-engage cognitive resources while walking in relatively easier secondary tasks. This dissociation between neural and motor responses underscores a PD-specific adaptation mechanism and highlights the importance of considering task complexity in understanding and managing dual-task interference in this population.

## Data Availability

Data will be provided by the corresponding author upon request.
